# Longing for better ultrasound-guided subclavian/axillary venous cannulation

**DOI:** 10.1186/s13054-018-2075-0

**Published:** 2018-06-05

**Authors:** Gentle Sunder Shrestha

**Affiliations:** Department of Anaesthesiology, Critical Care Unit, Tribhuvan University Teaching Hospital, Maharajgunj, Kathmandu, Nepal

Ultrasound guidance for central venous catheter (CVC) placement reduces complications, increases safety and enhances the quality of the procedure [1]. Existing guidelines suggest a higher level of evidence for ultrasound-guided internal jugular venous cannulation compared with the subclavian/axillary route [2]. Meta-analysis of trials studying the use of ultrasound guidance for subclavian venous cannulation, when compared with the landmark technique, showed decreased incidence of arterial puncture and hematoma formation, but there was no overall difference in complications and success rates [3].

Smaller studies have shown that patient positioning during cannulation of the subclavian vein (e.g., turning the head to the contralateral side, shoulder-pulled-downward position, and abducted position of arm) can alter the relationship between the subclavian vein and the internal jugular vein and can also alter the cross-sectional area of the vein, thus potentially affecting the safety and success of cannulation [4, 5]. Further well designed studies should try to explore the influence of these various patient positions on ultrasound-guided subclavian venous cannulation.

## Abbreviation

CVC: Central venous catheter
